# COVID-MATCH65—A prospectively derived clinical decision rule for severe acute respiratory syndrome coronavirus 2

**DOI:** 10.1371/journal.pone.0243414

**Published:** 2020-12-09

**Authors:** Jason A. Trubiano, Sara Vogrin, Olivia C. Smibert, Nada Marhoon, Adrian A. Alexander, Kyra Y. L. Chua, Fiona L. James, Nicholas R. L. Jones, Sam E. Grigg, Cecilia L. H. Xu, Nasreen Moini, Sam R. Stanley, Michael T. Birrell, Morgan T. Rose, Claire L. Gordon, Jason C. Kwong, Natasha E. Holmes

**Affiliations:** 1 Department of Infectious Diseases, Austin Health, Heidelberg, Australia; 2 Department of Medicine, Austin Health, University of Melbourne, Heidelberg, Australia; 3 Department of Infectious Diseases and The National Centre for Infections in Cancer, Peter MacCallum Cancer Centre, Melbourne, Australia; 4 Department of Medicine (St Vincent’s Hospital), University of Melbourne, Fitzroy, Australia; 5 Data Analytics Research and Evaluation (DARE) Centre, Austin Health and University of Melbourne, Heidelberg, Australia; 6 Department of General Medicine, Austin Health, Heidelberg, Australia; 7 Electronic Medical Record and Information and Communications Technology Services, Austin Health, Heidelberg, Australia; 8 Department of Microbiology and Immunology, Peter Doherty Institute for Infection and Immunity, University of Melbourne, Melbourne, Australia; Translational Medical Research Institute, Shanghai Public Health Clinical Center, Fudan University, CHINA

## Abstract

**Objectives:**

We report on the key clinical predictors of severe acute respiratory syndrome coronavirus 2 (SARS-CoV-2) infection and present a clinical decision rule that can risk stratify patients for COVID-19.

**Design, participants and setting:**

A prospective cohort of patients assessed for COVID-19 at a screening clinic in Melbourne, Australia. The primary outcome was a positive COVID-19 test from nasopharyngeal swab. A backwards stepwise logistic regression was used to derive a model of clinical variables predictive of a positive COVID-19 test. Internal validation of the final model was performed using bootstrapped samples and the model scoring derived from the coefficients, with modelling performed for increasing prevalence.

**Results:**

Of 4226 patients with suspected COVID-19 who were assessed, 2976 patients underwent SARS-CoV-2 testing (n = 108 SARS-CoV-2 positive) and were used to determine factors associated with a positive COVID-19 test. The 7 features associated with a positive COVID-19 test on multivariable analysis were: **C**OVID-19 patient exposure or international travel, **M**yalgia/malaise, **A**nosmia or ageusia, **T**emperature, **C**oryza/sore throat, **H**ypoxia–oxygen saturation < 97%, **65** years or older—summarized in the mnemonic **C**OVID-**MATCH65**. Internal validation showed an AUC of 0.836. A cut-off of ≥ 1.5 points was associated with a 92.6% sensitivity and 99.5% negative predictive value (NPV) for COVID-19.

**Conclusions:**

From the largest prospective outpatient cohort of suspected COVID-19 we define the clinical factors predictive of a positive SARS-CoV-2 test. The subsequent clinical decision rule, COVID-MATCH65, has a high sensitivity and NPV for SARS-CoV-2 and can be employed in the pandemic, adjusted for disease prevalence, to aid COVID-19 risk-assessment and vital testing resource allocation.

## Introduction

The COVID-19 pandemic caused by severe acute respiratory syndrome coronavirus 2 (SARS-CoV-2) was first reported in China and has now infected over 9 million people globally [1]. A range of clinical symptoms and syndromes have been reported in confirmed COVID-19 [2–4]. However, there have been limited prospective reports of the clinical and epidemiological predictors of COVID-19 infection [5]. We report on the clinical and epidemiological predictors of COVID-19 from a uniquely derived prospective database and present a point-of-care ready COVID-19 clinical decision tool.

## Methods

A COVID-19 rapid assessment screening clinic was established at Austin Health on 11 March 2020 with prospective electronic medical record (EMR; **S1 Data** and **S1 File**) data of patients presenting to the clinic systematically collected by medical staff from 11 March to 22 April 2020. Patients were predominantly adults—children over 6 months were seen at clinician discretion. Modifications to the EMR were made during the study period to align with the Victorian Department of Health and Human Services (DHHS) testing criteria [6] (**S1 Data** and **S1 File**). Only those patients that met the DHHS criteria for SARS-CoV-2 testing had nasopharyngeal swab collected for SARS-Cov-2 nucleic acid detection by polymerase chain reaction (PCR), the platforms utilized outlined in **S1 Data** and **S1 File**. Patients with swabs that had SARS-CoV-2 nucleic acid detected were termed “COVID-19 test positive”; those with swabs where SARS-CoV-2 nucleic acid was not detected were termed “COVID-19 test negative”. This study was approved by the Austin Health Human Research and Ethics Committee (Austin HREC Audit/20/Austin/37). Participants were not recruited and did not required to provide consent for this study as this was an audit of routine clinical practice and standardised data collection.

### Derivation and internal validation cohort

Clinical data from the data collection tool (baseline demographics, clinical symptoms, clinical observations) and COVID-19 testing results were extracted from Austin Health EMR platform (Cerner®) by the Data Analytics Research and Evaluation (DARE) Centre (Austin Health/University of Melbourne).

### Statistical analysis

All results are presented according to TRIPOD guidelines [7]. Categorical variables are presented as frequency (percentage) and continuous variables as median (interquartile range [IQR]). Fisher’s exact test or rank sum test were used to compare characteristics between tested and not tested patients. To determine the predictors of a positive COVID-19 test, a multivariable logistic regression with backward stepwise procedure was used, eliminating variables with p>0.10 and re-inclusion of variables with p<0.05. Bootstrapping was used for internal validation. Further details on variable selection, model development and performance, internal validation and score derivation are outlined in **eMethods 3 of S1 File**.

## Results

### Study population and setting

During the study period 4359 assessments were performed in 4226 patients (**S1 Table and S1 Fig of S1 File**). For those with multiple presentations (n = 118) only their first testing date was used (for patients that were not tested, their first assessment was taken). Median (IQR) number of daily assessments was 96 (71, 134) with an average of 51% of patients being tested each day (**S2 Fig of S1 File**).

The characteristics of those with suspected COVID-19 that presented to a COVID-19 testing service, stratified by testing performed status, is outlined in **S2 Table of S1 File.** The most frequently reported symptoms in both groups were any fever (reported or documented), cough, sore throat and coryza as outlined in **S2 Table of S1 File**. SARS-CoV-2 testing was undertaken in 2935patients (70%).

### COVID-19 test positivity

Of the 2976 patients that were tested, 41 were excluded from the analysis due to pending results (n = 38) or indeterminate results (n = 3) (**eFig 1 of S1 File**). The prevalence of a positive COVID-19 test in the final cohort was 3.7% (108/2935). Characteristics of those patients with a positive COVID-19 test are shown in **Table 1**.

**Table 1:** Characteristics of patients who underwent testing for COVID-19.

Factor		Overall	Not detected	Detected
N		2935	2827	108
Age, years, median (IQR)		39 (29, 53)	38 (29, 52)	51 (33, 62)
Sex—male		1071 (36.5%)	1016 (35.9%)	55 (50.9%)
Comorbidities				
	Cardiovascular disease	105 (3.6%)	101 (3.6%)	4 (3.7%)
	Diabetes	85 (2.9%)	84 (3.0%)	1 (0.9%)
	Hypertension	262 (8.9%)	245 (8.7%)	17 (15.7%)
	ACEI/ARB treatment	98 (3.3%)	89 (3.1%)	9 (8.3%)
	Smoking	259 (8.8%)	256 (9.1%)	3 (2.8%)
	Chronic renal or liver disease	21 (0.7%)	21 (0.7%)	0 (0.0%)
	Immunosuppressed	90 (3.1%)	87 (3.1%)	3 (2.8%)
	Chronic respiratory disease	343 (11.7%)	339 (12.0%)	4 (3.7%)
Pregnancy		38 (1.3%)	38 (1.3%)	0 (0.0%)
Overseas health facility exposure		114 (3.9%)	112 (4.0%)	2 (1.9%)
Australian health facility exposure		902 (30.7%)	890 (31.5%)	12 (11.1%)
Any contact or overseas travel		1182 (40.3%)	1093 (38.7%)	89 (82.4%)
Contact with known COVID-19 positive patient		508 (17.3%)	446 (15.8%)	62 (57.4%)
Overseas travel (incl. cruise)		723 (24.6%)	684 (24.2%)	39 (36.1%)
Days from arrival to symptom onset, median (IQR)		2 (-1, 6)	2 (-1, 6)	1 (-1, 3)
Number of symptoms				
	0	49 (1.7%)	45 (1.6%)	4 (3.7%)
	1	243 (8.3%)	240 (8.5%)	3 (2.8%)
	2	540 (18.4%)	526 (18.6%)	14 (13.0%)
	3	669 (22.8%)	646 (22.9%)	23 (21.3%)
	4	646 (22.0%)	623 (22.0%)	23 (21.3%)
	5 or more	788 (26.8%)	747 (26.4%)	41 (38.0%)
Symptoms				
	Any fever	1119 (38.1%)	1063 (37.6%)	56 (51.9%)
	Fever > 38 C	274 (9.3%)	260 (9.2%)	14 (13.0%)
	Fever subjective	905 (30.8%)	859 (30.4%)	46 (42.6%)
	Sore throat	2038 (69.4%)	1983 (70.1%)	55 (50.9%)
	Sinusitis	14 (0.5%)	13 (0.5%)	1 (0.9%)
	Cough	2042 (69.6%)	1956 (69.2%)	86 (79.6%)
	Shortness of breath	897 (30.6%)	868 (30.7%)	29 (26.9%)
	Chest pain	71 (2.4%)	68 (2.4%)	3 (2.8%)
	Anosmia	75 (2.6%)	64 (2.3%)	11 (10.2%)
	Ageusia	81 (2.8%)	69 (2.4%)	12 (11.1%)
	Anosmia or ageusia	126 (4.3%)	109 (3.9%)	17 (15.7%)
	Coryza	1606 (54.7%)	1559 (55.1%)	47 (43.5%)
	Diarrhoea	483 (16.5%)	457 (16.2%)	26 (24.1%)
	Other GI symptoms	63 (2.1%)	62 (2.2%)	1 (0.9%)
	Malaise/myalgia/arthralgia	1410 (48.0%)	1339 (47.4%)	71 (65.7%)
	Headache	402 (13.7%)	381 (13.5%)	21 (19.4%)
	Asymptomatic	25 (0.9%)	23 (0.8%)	2 (1.9%)
Days since symptom onset, median (IQR)		3 (1, 6)	3 (1, 6)	4 (2, 7)
Clinical signs				
	SPO2, median (IQR)	98 (97, 99)	98 (97, 99)	98 (96, 99)
	Temperature Tympanic, median (IQR)	36.6 (36.3, 36.9)	36.6 (36.3, 36.9)	36.7 (36.3, 37.1)
	Systolic Blood Pressure, median (IQR)	133 (121, 147)	132 (121, 147)	134 (122, 146)
	Diastolic Blood Pressure, median (IQR)	82 (75, 89)	81 (75, 89)	83.5 (78, 88)
	Respiratory Rate, median (IQR)	18 (16, 18)	18 (16, 18)	18 (17, 18)
	Pulse Rate, median (IQR)	83 (73, 94)	84 (73, 94)	82 (73, 93.5)
Discharge destination				
	Discharged	1895 (64.6%)	1802 (63.7%)	93 (86.1%)
	Transferred to ED	18 (0.6%)	18 (0.6%)	0 (0.0%)
	Transferred to ward	1 (<0.1%)	1 (<0.1%)	0 (0.0%)
	Unknown	1021 (34.8%)	1006 (35.6%)	15 (13.9%)

**Abbreviations:** N, number; IQR, interquartile range; SPO2, oxygen saturation; ACEI, angiotensin-converting-enzyme inhibitor; ARB, angiotensin receptor blocker; GI, gastrointestinal; ED, emergency department.

### Demographic, epidemiological and clinical factors associated with a positive COVID-19 test

**Fig 1:**
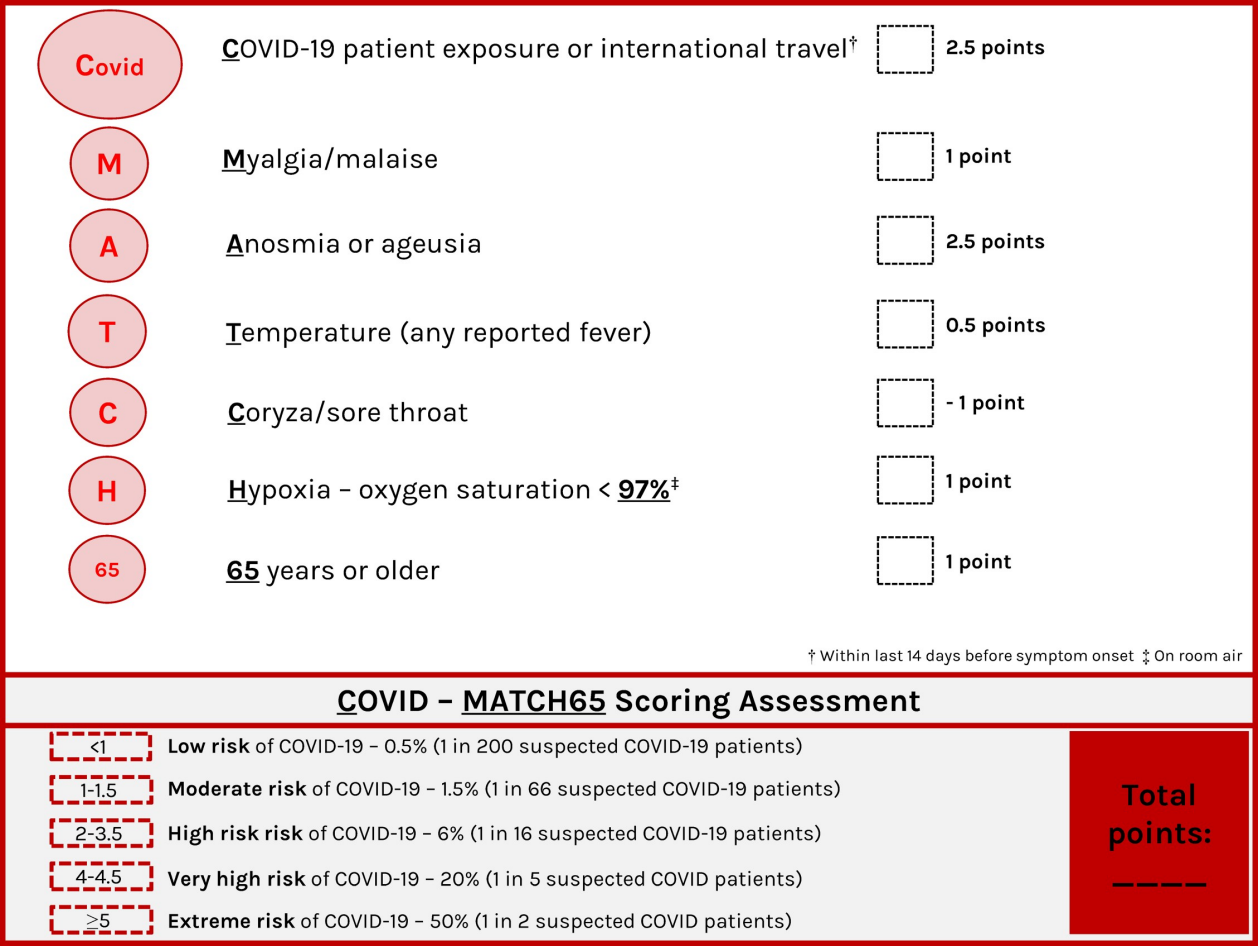
COVID-19 clinical decision rule–COVID-MATCH65.

The characteristics associated with a positive COVID-19 test in univariate and multivariable analysis are shown in **Table 2**. The seven features associated with a COVID-19 test on multivariable analysis were summarized in the mnemonic COVID-MATCH65 (**Fig 1**). The model showed good discrimination (AUC = 0.843, Hosmer-Lemeshow chi^2^ = 4.96, p = 0.762) and calibration (calibration slope = 1.00, Brier score = 0.03, product-moment correlation between observed and predicted probability = 0.35). Internal validation showed minimal mean optimism of 0.007 with internally validated AUC of 0.836 **(S3 & S4 Figs of S1 File).** The resulting score ranges from -1 to 6.5 points with score ≤ 1 representing low risk of a positive test (<1%) and scores above 4 having beyond 20% probability of a positive test (**Fig 1**).

**Table 2:** Univariate & multivariable analysis of features associated with a positive COVID-19 test (SARS-CoV-2 nucleic acid detected).

Variables considered for inclusion	Overall tested (n = 2935)	COVID-19 positive test (n = 108)	Univariate analysis		Multivariable analysis				
			OR (95% CI)	p-value	OR (95% CI)	Beta coefficient (95% CI)	p-value	Presence in 1000 bootstrap replications, %*	Points
Age 65+	254 (8.7%)	19 (17.6%)	2.35 (1.41, 3.93)	0.001	2.80 (1.56, 5.04)	1.03 (0.45, 1.62)	0.001	75	1
Male sex	1071 (36.5%)	55 (50.9%)	1.85 (1.26, 2.72)	0.002				50	
Hypertension	262 (8.9%)	17 (15.7%)	1.97 (1.15, 3.36)	0.013				50	
Contact with known COVID-19 positive patient or overseas travel	1182 (40.3%)	89 (82.4%)	7.43 (4.50, 12.27)	<0.001	14.24 (7.92, 25.63)	2.66 (2.07, 3.24)	<0.001	100	2.5
Any fever (documented or reported)	1119 (38.1%)	56 (51.9%)	1.79 (1.22, 2.63)	0.003	1.59 (1.03, 2.43)	0.46 (0.03, 0.89)	0.035	71	0.5
Coryza or sore throat	2455 (83.6%)	73 (67.6%)	0.39 (0.26, 0.59)	<0.001	0.36 (0.23, 0.58)	-1.01 (-1.48, -0.55)	<0.001	99	-1
Cough	2042 (69.6%)	86 (79.6%)	1.74 (1.08, 2.80)	0.022				52	
Shortness of breath*	897 (30.6%)	29 (26.9%)	0.83 (0.54, 1.28)	0.394					
Anosmia or ageusia	126 (4.3%)	17 (15.7%)	4.66 (2.68, 8.09)	<0.001	13.67 (6.89, 27.13)	2.62 (1.93, 3.30)	<0.001	100	2.5
Diarrhoea	483 (16.5%)	26 (24.1%)	1.64 (1.05, 2.58)	0.031				26	
Myalgia or Malaise	1410 (48.0%)	71 (65.7%)	2.13 (1.42, 3.19)	<0.001	2.20 (1.41, 3.44)	0.79 (0.45, 1.35)	0.001	96	1
Headache	402 (13.7%)	21 (19.4%)	1.55 (0.95, 2.53)	0.079				36	
SPO2 <97%	473 (16.1%)	36 (33.3%)	2.73 (1.81, 4.13)	<0.001	2.46 (1.57, 3.87)	0.90 (0.45, 1.35)	<0.001	93	1
Temperature ≥37.5 C	174 (5.9%)	11 (10.2%)	1.85 (0.97, 3.53)	0.060				15	
Systolic blood pressure >140 mmHg*	1082 (36.9%)	43 (39.8%)	1.14 (0.77, 1.69)	0.518					
Diastolic blood pressure >80 mmHg	1623 (55.3%)	72 (66.7%)	1.65 (1.10, 2.47)	0.016				54	
Respiratory rate <16/min or >20/min*	196 (6.7%)	7 (6.5%)	0.97 (0.44, 2.11)	0.934					
Pulse rate <60/min or >100/min	486 (16.6%)	11 (10.2%)	0.56 (0.30, 1.06)	0.073				51	

*Not considered for inclusion due to p<0.200.

**Abbreviations:** OR, odds ratio; CI, confidence interval; SPO2, oxygen saturation.

The positive and negative results for each COVID-MATCH65 score are outlined in **S3 Table of S1 File**. A score of at least 1.5 was shown to have 92.6% (95% CI 85.9%, 96.7%) sensitivity, 51.3% (49.4, 53.1) specificity, 6.8% (5.5, 8.2) positive predictive value and 99.5% (98.9, 99.8) negative predictive value of identifying a patient who was COVID-19 test positive (**S4 Table of S1 File**). COVID-MATCH65 also retains a high NPV with increasing prevalence of COVID-19 (30% prevalence) (**S3 Table of S1 File**).

### Admission to hospital

A total of 15 COVID-19 positive patients (14%) were admitted to hospital. Median (IQR) COVID-MATCH65 score in admitted was 3.5 (2.5, 4.5) while in non-admitted it was 3 (2.5, 4). Score was not predictive of admission (OR 1.04, 95%CI: 0.70, 1.53, p = 0.852). Variables predictive of admission were oxygen saturation (SpO2) < 97%, shortness of breath, male gender and not being exposed to confirmed case/international travel (**S5 Table of S1 File**).

## Discussion

Whilst the clinical features of COVID-19 have been reported, robust prospective data from patients presenting for COVID-19 assessment (SARS-CoV-2 positive and negative) remains absent. Therefore, the clinical predictors associated with a positive SARS-CoV-2 test have remained ill defined. Whilst fever has been the predominant presenting feature of confirmed COVID-19 cases from published inpatient populations [4], it was in fact observed less frequently (36.5%) in our outpatient cohort, potentially the result of earlier presentation (5 days[median] from symptom onset). Bajema *et al*. [5] reported fever in 68% in a retrospective cohort study (n = 210) from the USA with similar incidence rate of COVID-19 positive tests to our cohort (5% USA vs. 4.7% AUS). Whilst in the earliest reports from confirmed cases in China the figures were 83–98% [2, 3]. Coryza and sore throat were also frequently reported, the presence of either was in fact a negative predictor of COVID-19. Anosmia or ageusia as seen in other emerging studies was a strong predictor of a positive COVID-19 test [8]. Whilst contact and/or international travel was a predictor of COVID-19 infection in our model, as seen in US model from Challenger *et al*. [9], it may be less relevant in outbreak settings and during periods of travel bans, however these criteria alone are not required for a patient to be at high risk of COVID-19.

Our model has some limitations, including the single centre prospective data source, jurisdictional guided testing criteria, testing of symptomatic only patients and absence of external validation. However, only one small retrospective US cohort (n = 49 COVID-19 positive /n = 98 COVID-19 negative) [9] and two non-peer reviewed publications from China have examined the role of clinical decision rules from large datasets—Meng *et al*. (n = 620 outpatients; 48.7% positive) [10] and Song *et al*. [11] (n = 304 inpatients; 24.0% positive), both limited by requirement for clinical and laboratory parameters. COVID-MATCH65 uses readily available clinical information without laboratory test results, with a score of ≥ 1.5 associated with high sensitivity (92.6 [95% CI 85.9, 96.7]) and NPV (99.5 [95% CI 98.9, 99.8]), enabling application in the outpatient and potentially early inpatient setting. The model also retains a high NPV (99.3 [95% CI 98.9, 99.6]) with a score of ≥ 2 with only a slightly reduced sensitivity, which may appeal to some centres trying to reduce unnecessary testing. Further risk stratification can be made with the COVID-MATCH65 (lowest risk [< 1 in 100] to extreme risk [1 in 1]), aiding diagnostic approaches in patients with suspected COVID-19, such as additional testing or serological evaluation With ranging SARS-CoV-2 prevalence internationally, it is important to note that COVID-MATCH65 also performed well with increasing disease prevalence (Table 3). In a pandemic where diagnostic resources are limited in both low- and high-income settings, [12] risk stratification of those likely to have COVID-19 is urgently required and tools such as COVID-MATCH65 can aid the front-line clinician. We encourage readers to urgently employ and validate COVID-MATCH65 in their own datasets, as it is likely to aid clinicians at point-of-care especially via an open access web platform (http://COVID-MATCH65.austin.org.au).

**Table 3:** The sensitivity, specificity, positive predictive value and negative predictive value of COVID-MATCH65 with increasing prevalence of COVID-19.

Sensitivity (%)	Specificity (%)	Prevalence (%)	PPV	NPV
93	35	4	5.0	99.4
93	35	5	7.1	99.1
93	35	10	13.8	98.1
93	35	20	26.6	95.9
93	35	30	38.3	93.2
93	35	40	49.1	89.7
93	35	50	59.1	85.4
93	35	60	68.4	79.5
93	35	70	77.1	71.4
93	35	80	85.3	59.3

## Supporting information

S1 Data (XLSX)

S1 File (PDF)
