# Supplementary material for: COVID-MATCH65—A prospectively derived clinical decision rule for severe acute respiratory syndrome coronavirus 2
Source: PLoS One. 2020 Dec 9;15(12):e0243414. doi: 10.1371/journal.pone.0243414 (PMC7725390; doi:10.1371/journal.pone.0243414)
Supplement: S1 File — (PDF) [file pone.0243414.s002.pdf]

S1 File

eMethods 1: Data Collection Tool 2

eMethods 2: Victorian DHHS criteria for eligibility for SARS-CoV-2 testing over time.......4

eMethods 3: SARS-CoV-2 Testing 12

eMethods 4: Statistical Analysis 12

eResults – Tables and Figures 14

eTable 1: Baseline characteristics of total cohort assessed in COVID-19 screening clinic... 14

eTable 2: Baseline characteristics of suspected cases 16

eTable 3 – Positive and negative results for COVID-MATCH65 18

eTable 4: The sensitivity, specificity, positive predictive value and negative predictive value with 95% confidence intervals of various cut-off levels of COVID-MATCH65 18

eFigure 1: Participants assessed and tested in this study 20

eFigure 2: Number of COVID-19 assessments performed per day 21

eFigure 3: Area under the receiver operating characteristic curve (AUC) analysis of COVID-MATCH65 22

eFigure 4: Calibration of the COVID-MATCH65 23

References 24

eMethods 1: Data Collection Tool

Attach patient label here

CLINICAL ASSESSMENT FOR COVID-19 CLINIC

CLINICAL ASSESSMENT				
**1. Clinical observations**			**Record in Cerner**	
**2. Do you have any of the following symptoms?**				
☐ Fever (>38° C)		☐ Shortness of breath	☐ Cough	☐ Anosmia
☐ Fever (subjective)		☐ Sore throat	☐ Coryzal symptoms	
☐ Diarrhoea	☐ Malaise/myalgia	☐ Asymptomatic	☐ Other:	
**3. Date of first symptom onset?**			**Date:**	

**4. Do you have any medical comorbidities**		☐ YES		☐ NO
☐ Cardiac disease	☐ Diabetes	☐ HTN	☐ ACEI/ARB	☐ Smoking
☐ Chronic liver or renal disease	☐ Immunosuppressed (e.g. cancer, transplant)		☐ Chronic resp disease (e.g. COPD, Asthma)	☐ Pregnancy
**5. In the last 28 days, have you:**				
Had contact with a case of COVID-19 (coronavirus)?		☐ YES		☐ NO
**6. In the last 28 days, have you:**				
Been in a prison, jail or military barracks?		☐ YES		☐ NO
**7. In the last 28 days, have you:**				
Travelled on cruise ship?		☐ YES		☐ NO
**8. Do you identify as an Aboriginal or Torres Strait Islander?**		☐ YES		☐ NO

**9. Have you been in or transited through any other countries at any time in the last 28 days?**		☐ YES	☐ NO
Please list countries visited		Date arrived	Date departed
**10. When did you arrive back in Australia?**			
Date:	Airline:	Flight number:	

**11. In the last 28 days, have you:**		
Visited/Attended/Worked in a healthcare facility/hospital overseas?	☐ YES	☐ NO
Worked in an Australian healthcare system?	☐ YES	☐ NO

Attach patient label here

RISK ASSESSMENT

1: Travel overseas or cruise ship OR 2: Contact with coronavirus	Symptoms (FEVER > 38 or COUGH or SOB or SORE THROAT) Within 14 days of travel	Action
☐ YES	☐ YES	• Test for COVID-19 – nasopharyngeal (red) swab OR nose + throat (green) swab<br>• Assess if patient requires admission?
☐ YES	☐ NO	• If overseas or cruise ship travel OR contact with coronavirus – home quarantine until 14 days after travel / contact<br>• Repeat testing only if symptomatic
☐ NO	☐ YES	• Manage as per usual (**Testing for COVID-19 ONLY in healthcare worker, aged care resident, prison/jail/military or ABTSI**)
☐ NO	☐ NO	• Discharge, nil further

OUTCOME CHECKLIST

Swab taken	Return to ED	Patient Information given	Inpatient Admission
☐ Yes<br>☐ Not required	☐ Yes<br>☐ No	☐ Yes<br>☐ No	☐ Yes<br>☐ Not required

Is there a suspected alternative infective diagnosis?			☐ Yes	☐ No
☐ URTI (non COVID19)	☐ UTI	☐ Cellulitis	☐ Gastro	☐ Fever F.I**
☐ Other (provide details):				

If fever F.I. in return traveller please speak with infectious diseases OR if further support required: **Infectious Diseases via switch for advice when Supervising Consultant not present.

Any additional comments? :

FOR COMPLETION SIGN OFF

Doctor	Nurse
Date:<br>Name:<br>Signature:	Date:<br>Name:<br>Signature:

Note – This is the tool utilized on last day of data collection 7th April 2020. Earlier versions were subsequently modified to reflect DHHS up to date testing criteria and additional past medical history or symptomatology that were frequently reported (i.e. hypertension, ACE inhibitor utilization, anosmia).

Abbreviations: ED; emergency department; URTI; upper respiratory infection; UTI; urinary tract infection; Gastro, gastroenteritis; F.I., for investigation.

eMethods 2: Victorian DHHS criteria for eligibility for SARS-CoV-2 testing over time

Date	Criteria
23 January 2020	Both clinical and epidemiological criteria need to be met for a person to be classified as a suspected case.<br>Clinical criteria:<br>Fever or history of fever<br>AND<br>Acute respiratory infection (shortness of breath or cough or sore throat)<br>OR<br>Severe acute respiratory infection without fever requiring hospitalisation<br>AND<br>Epidemiological criteria:<br>A history of being in a location designated by the department as a place where there is evidence of human to human transmission (see below) in the 14 days prior to symptom onset<br>OR<br>Close contact within 14 days of symptom onset with any of the following:<br>• a confirmed or suspected case of 2019-nCoV;<br>• a healthcare facility in a country where hospital-associated infections have been reported.<br>As of 23 January 2020 the declared locations are:<br>Wuhan City, Hubei Province, China.
29 January 2020	As above<br>OR<br>A patient with severe acute respiratory infection (SARI) and a history of travel to any part of China in the 14 days prior to symptom onset, after discussion with department, may be classified as a suspected case and tested for novel coronavirus. As per the World Health Organization definition SARI is an illness with fever AND cough AND admission to hospital<br>OR<br>A casual contact with compatible symptoms, after discussion with the department, may be classified as a suspected case and tested for novel coronavirus.<br>As of 29 January 2020 the declared locations are:<br>Hubei Province, China, including Wuhan City
4 February 2020	Both clinical and epidemiological criteria need to be met for a person to be classified as a suspected case.<br>Clinical criteria:<br>Fever<br>OR<br>Acute respiratory infection (for example, shortness of breath or cough)<br>AND

	Epidemiological criteria:<br>A history of being in mainland China*<br>OR<br>Having close contact with a confirmed case of 2019-nCoV in the 14 days prior to symptom onset.<br>OR<br>A casual contact with compatible symptoms, after discussion with the department, may be classified as a suspected case and tested for novel coronavirus.<br>Notes:<br>*Mainland China excludes Hong Kong, Macau and Taiwan
8 February 2020	Both clinical and epidemiological criteria need to be met for a person to be classified as a suspected case.<br>Clinical criteria:<br>Fever<br>OR<br>Acute respiratory infection (for example, shortness of breath or cough) with or without fever<br>AND<br>Epidemiological criteria:<br>Travel to (including transit through) mainland China* in the 14 days before onset of illness<br>OR<br>Close or casual contact with a confirmed case of 2019-nCoV in the 14 days before onset of illness.<br>Notes:<br>*Mainland China excludes Hong Kong, Macau and Taiwan
11 February 2020	Both clinical and epidemiological criteria need to be met for a person to be classified as a suspected case.<br>Clinical criteria:<br>Fever<br>OR<br>Acute respiratory infection (for example, shortness of breath or cough) with or without fever<br>AND<br>Epidemiological criteria:<br>Travel to (including transit through) mainland China* in the 14 days before onset of illness<br>OR<br>Close or casual contact with a confirmed case of 2019-nCoV in the 14 days before onset of illness.<br>Notes:<br>*Mainland China excludes Hong Kong, Macau and Taiwan<br>Testing for 2019-nCoV in people who have not travelled to mainland China within the previous 14 days is now at the discretion of the treating clinician. As of 11 February, there are an increasing number of countries where there may be community transmission of novel coronavirus. When a patient has an illness that is compatible with novel coronavirus infection and has travelled overseas, testing can be considered. Call the department for advice on whether testing should be actively considered.

15 February 2020	Both clinical and epidemiological criteria need to be met for a person to be classified as a suspected case.<br>Clinical criteria:<br>Fever<br>OR<br>Acute respiratory infection (for example, shortness of breath or cough) with or without fever<br>AND<br>Epidemiological criteria:<br>Travel to (including transit through) mainland China* in the 14 days before the onset of illness<br>OR<br>Close or casual contact in the 14 days before illness onset with a confirmed case of COVID-19.<br>Notes:<br>*Mainland China excludes Hong Kong, Macau and Taiwan<br>It is recommended that clinicians consider testing people with a clinically compatible illness who travelled to any of the following countries in the 14 days before onset of symptoms:<br>• Hong Kong<br>• Indonesia<br>• Japan<br>• Singapore<br>• Thailand<br>This list is based on the volume of travel between those countries, Australia and China, and/or the current epidemiology of COVID-19; however, the risk of COVID-19 in these countries is currently thought to be low. Clinical and public health judgement should be applied. The recommendation does not apply to passengers who have only been in transit through an airport in these countries.
3 March 2020	A. If the patient satisfies both clinical and epidemiological criteria, they are classified as a suspected case:<br>Clinical criteria:<br>Fever<br>OR<br>Acute respiratory infection (for example, shortness of breath or cough) with or without fever<br>AND<br>Epidemiological criteria:<br>Travel to (including transit through) a country considered to pose a risk of transmission* in the 14 days before onset of illness<br>OR<br>Close or casual contact in the 14 days before illness onset with a confirmed case of COVID-19.<br>B. If the patient has severe community-acquired pneumonia (critically ill) and no other cause is identified, with or without recent international travel, they are classified as a suspected case.

	C. If the patient has moderate or severe community-acquired pneumonia (hospitalised) and is a healthcare worker, with or without international travel, they are classified as a suspected case.<br>Notes:<br>*Country transmission risk assessment<br>Higher risk: Mainland China (excludes Hong Kong, Macau and Taiwan), Iran, Italy, South Korea<br>Moderate risk: Cambodia, Japan, Hong Kong, Indonesia, Singapore, Thailand<br>This list is based on the risk of the person having been exposed to COVID-19 due to travel to a country with sustained community transmission and other epidemiological evidence.
9 March 2020	A. If the patient satisfies both clinical and epidemiological criteria, they are classified as a suspected case:<br>Clinical criteria:<br>Fever<br>OR<br>Acute respiratory infection (for example, shortness of breath or cough) with or without fever<br>AND<br>Epidemiological criteria:<br>International travel in the 14 days before the onset of illness<br>OR<br>Close or casual contact in the 14 days before illness onset with a confirmed case of COVID-19.<br>B. If the patient has severe community-acquired pneumonia (critically ill*) and no other cause is identified, with or without recent international travel, they are classified as a suspect case.<br>If the patient has moderate or severe community-acquired pneumonia (hospitalised) and is a healthcare worker, with or without international travel, they are classified as a suspect case.
15 March 2020	A. If the patient satisfies both clinical and epidemiological criteria, they are classified as a suspected case:<br>Clinical criteria:<br>Fever<br>OR<br>Acute respiratory infection (for example, shortness of breath or cough) with or without fever<br>AND<br>Epidemiological criteria:<br>International travel in the 14 days before the onset of illness<br>OR<br>Close contact in the 14 days before illness onset with a confirmed case of COVID-19<br>B. If the patient has severe community-acquired pneumonia (critically ill*) and no other cause is identified,

	with or without recent international travel, they are classified as a suspected case.<br>*Critically ill means requiring care in ICU/HDU, or for patients in which ICU care is not appropriate, respiratory or multiorgan failure. Clinical judgement should be exercised considering the likelihood of COVID-19.<br>C. If any healthcare worker with direct patient contact, residential aged care worker or aged care resident has a fever (≥ 37.5) AND an acute respiratory infection (e.g. shortness of breath, cough, sore throat), they are classified as a suspect case.
20 March 2020	Patients who meet at least one clinical AND at least one epidemiological criteria should be tested.<br>Clinical criteria:<br>Fever*<br>OR<br>Acute respiratory infection (for example, shortness of breath, cough, sore throat)<br>Epidemiological criteria:<br>Travelers from overseas with onset of symptoms within 14 days of return<br>OR<br>Close contacts of confirmed COVID-19 cases with onset of symptoms within 14 days of last contact<br>OR<br>Healthcare workers and residential aged care workers meeting clinical criteria<br>OR<br>Aged and residential care residents meeting clinical criteria<br>OR<br>Patients who are Aboriginal or Torres Strait Islander people meeting clinical criteria<br><br>The following patients should also be tested:<br>Patients admitted to hospital with acute respiratory tract infection AND fever*<br>Notes:<br>* ≥38 degrees, without another immediately apparent cause such as urinary tract infection or cellulitis
1 April 2020	Patients who meet at least one clinical AND at least one epidemiological criterion should be tested.<br>Clinical criteria:<br>Fever (≥38°C) or history of fever (for example night sweats, chills)<br>OR<br>Acute respiratory infection (for example, shortness of breath, cough, sore throat)<br>Epidemiological criteria:<br>Close contacts of confirmed COVID-19 cases with onset of symptoms within 14 days of last contact<br>OR

	Travelers from overseas with onset of symptoms within 14 days of return<br>OR<br>Cruise ship passengers and crew with onset of symptoms within 14 days of return<br>OR<br>Paid or unpaid workers in healthcare, residential care, and disability care settings<br>OR<br>People who have worked in public facing roles within homelessness support, child protection and the police force within the last 14 days<br>OR<br>Immunosuppressed patients admitted to hospital<br>People at any age with significant immunosuppression, as defined as:<br>• Haematologic neoplasms: leukemias, lymphomas, myelodysplastic syndromes<br>• Post-transplant: solid organ (on immunosuppressive therapy), haematopoietic stem cell transplant (within 24 months or on treatment for GVHD)<br>• Immunocompromised due to primary or acquired immunodeficiency (including HIV infection)<br>• Current chemotherapy or radiotherapy<br>• High-dose corticosteroids (≥20 mg of prednisone per day, or equivalent) for ≥14 days)<br>• All biologics and most disease-modifying anti-rheumatic drugs (DMARDs) as defined as follows:<br>o Azathioprime >3.0 mg/kg/day<br>o 6-Mercaptopurine >1.5 mg/kg/day<br>o Methotrexate >0.4 mg/kg/week<br>o Prednisone >20 mg/day. If <14 days treatment, can resume work when treatment ceased<br>o Tacrolimus (any dose)<br>o Cyclosporine (any dose)<br>o Cyclophosphamide (any dose)<br>o Mycophenolate (any dose)<br>o Combination (multiple) DMARDs irrespective of dose<br>OR<br>Patients who are Aboriginal or Torres Strait Islander people<br>OR<br>Patients in other high-risk settings<br><br>Note: High risk settings include:<br>• Aged care, disability and other residential care facilities<br>• Military operational settings<br>• Boarding schools<br>• Correctional facilities<br>• Detention centres

	• Settings where COVID-19 outbreaks have occurred, in consultation with the department<br>The following patients should also be tested:<br>Patients admitted to hospital with acute respiratory tract infection AND fever (≥38°C) without another immediately apparent cause such as urinary tract infection or cellulitis.
5 April 2020	Patients who meet at least one clinical AND at least one epidemiological criterion should be tested:<br>Clinical criteria:<br>Fever (≥38°C) or history of fever (for example night sweats, chills)<br>OR<br>Acute respiratory infection (for example, shortness of breath, cough, sore throat).<br>Epidemiological criteria:<br>Close contacts of confirmed COVID-19 cases with onset of symptoms within 14 days of last contact<br>OR<br>Travelers from overseas with onset of symptoms within 14 days of return<br>OR<br>Cruise ship passengers and crew with onset of symptoms within 14 days of disembarkation<br>OR<br>Paid or unpaid workers in healthcare, residential care, and disability care settings<br>OR<br>People who have worked in public facing roles in the following settings within the last 14 days:<br>• homelessness support<br>• child protection<br>• the police force<br>• firefighters who undertake emergency medical response<br>• childcare and early childhood education<br>• primary or secondary schools<br>OR<br>Any person aged 65 years or older<br>OR<br>Aboriginal or Torres Strait Islander peoples<br>OR<br>Patients admitted to hospital where no other cause is identified<br>OR<br>Any person in other high-risk settings, including:<br>• Aged care, disability and other residential care facilities<br>• Military operational settings<br>• Boarding schools<br>• Correctional facilities<br>• Detention centres

	• Settings where COVID-19 outbreaks have occurred, in consultation with the department.
14 April 2020	Patients who meet the following clinical criteria should be tested:<br>Fever OR chills in the absence of an alternative diagnosis that explains the clinical presentation<br>OR<br>Acute respiratory infection that is characterised by cough, sore throat or shortness of breath<br><br>Note: In addition, testing is recommended for people with new onset of other clinical symptoms consistent with COVID-19*AND who are close contacts of a confirmed case of COVID-19 or who have returned from overseas in the past 14 days.<br>* headache, myalgia, runny or stuffy nose, anosmia, nausea, vomiting, diarrhoea
27 April 2020	People without symptoms should not be tested expect in special circumstances such as recovered cases wishing to return to work in a healthcare facility or aged care facility or where requested by the department as part of outbreak management or enhanced surveillance.<br>Patients who meet the following clinical criteria should be tested:<br>Fever OR chills in the absence of an alternative diagnosis that explains the clinical presentation*<br>OR<br>Acute respiratory infection (e.g. cough, sore throat, shortness of breath, runny nose or anosmia)<br><br>Note: In addition, testing is recommended for people with new onset of other clinical symptoms consistent with COVID-19** AND who are close contacts of a confirmed case of COVID-19; who have returned from overseas in the past 14 days; or who are healthcare or aged care workers<br><br>*Clinical discretion applies including consideration of the potential for co-infection (e.g. concurrent infection with SARS-CoV-2 and influenza)<br>**headache, myalgia, stuffy nose, nausea, vomiting, diarrhoea

eMethods 3: SARS-CoV-2 Testing

There were three different assays in use during this period due to reagent shortages: AusDiagnostics Coronavirus Typing (8-well) assay, performed at Austin Pathology; in house E gene assay with primers based on Corman *et al.*(Corman et al., 2020) performed at the Microbiological Diagnostic Unit Public Health Laboratory; and in house RdRP primer assay and E gene assay based on Corman *et al.* (Corman et al., 2020) performed at the Victorian Infectious Diseases Reference Laboratory.

eMethods 4: Statistical Analysis

To determine the predictors of positive COVID-19 test univariate logistic regression was performed on patient's demographic characteristics, past medical history, exposure, clinical symptoms and signs. Variables with a low prevalence in positive COVID-19 group (<5%) were not included. Continuous variables were recoded into categorical variables using clinically relevant cut-off points. Categorical variables with large numbers of categories were recoded into smaller number of categories based on clinical experience and their associations with the outcome.

Variables with $p<0.20$ on univariate analysis were included in the multivariable logistic regression model. A backward stepwise procedure was used, eliminating variables with $p>0.10$ and re-inclusion of variables with $p<0.05$. This procedure was replicated in 1000 bootstrap samples and only variables present in at least 60% of replication were included in the final model.(Austin and Tu, 2004) Model performance in the original sample was evaluated by calculating C-statistic, Hosmer-Lemeshow statistic, calibration slope and Brier score.

Internal validation was performed by regular bootstrap procedure using 500 bootstrapped samples.(Steyerberg et al., 2001) Bootstrap performance of the final model was evaluated in each bootstrapped sample by calculating C-statistic and test performance of the same model in original sample. The optimism was calculated by subtracting test performance from bootstrap performance. Internally validated performance was calculated by subtracting mean optimism from apparent performance of the final model in the original sample.

A score for the final model was developed by rounding the coefficients of the logit model to the nearest half number. Predicted and observed risk were calculated for each score. Sensitivity,

specificity, area under the receiver operating characteristic curve (AUC), positive and negative predictive values (PPV, NPV) were calculated for each cut-off, and the final risk groups were chosen based on sensitivity and negative predictive value.

Logistic regression was used to evaluate the association between score and admission to hospital.

eResults – Tables and Figures

eTable 1: Baseline characteristics of total cohort assessed in COVID-19 screening clinic

**Factor**		**Value**
N		4226
Age, years, median (IQR)		39 (29, 54)
Sex - Male		1668 (39.5%)
Aboriginal and/or Torres Strait Islander		41 (1.0%)
Comorbidities		
	Cardiovascular disease	155 (3.7%)
	Diabetes	131 (3.1%)
	Hypertension	391 (9.3%)
	ACEI/ARB treatment	139 (3.3%)
	Smoking	397 (9.4%)
	Chronic renal or liver disease	37 (0.9%)
	Immunosuppressed	133 (3.1%)
	Chronic respiratory disease	462 (10.9%)
Pregnancy		56 (1.3%)
Overseas health facility exposure		124 (2.9%)
Australian health facility exposure		1030 (24.4%)
Any contact or overseas travel		1331 (31.5%)
Contact with confirmed COVID-19 case		567 (13.4%)
Overseas travel (incl. cruise)		815 (19.3%)
Days from arrival to symptom onset, median (IQR)		2 (-1, 6)
Number of reported symptoms		
	0	376 (8.9%)
	1	338 (8.0%)
	2	696 (16.5%)
	3	861 (20.4%)
	4	840 (19.9%)
	5 or more	1115 (26.4%)
Symptoms		
	Any reported fevers	1595 (37.7%)
	Fever > 38°C	389 (9.2%)
	Fever subjective	1298 (30.7%)
	Sore throat	2681 (63.4%)
	Sinusitis	20 (0.5%)
	Cough	2706 (64.0%)
	Shortness of breath	1256 (29.7%)
	Chest pain	111 (2.6%)
	Anosmia	102 (2.4%)
	Ageusia	120 (2.8%)
	Anosmia or ageusia	179 (4.2%)
	Coryza	2132 (50.4%)
	Diarrhoea	653 (15.5%)
	Other GI symptoms	92 (2.2%)
	Malaise/myalgia/arthralgia	1901 (45.0%)

	Headache	544 (12.9%)
	Asymptomatic	341 (8.1%)
Days since symptom onset, median (IQR)		3 (2, 7)
Clinical signs		
	SPO2, median (IQR)	98 (97, 99)
	Temperature Tympanic, median (IQR)	36.6 (36.3, 36.9)
	Systolic Blood Pressure, median (IQR)	132 (121, 147)
	Diastolic Blood Pressure, median (IQR)	82 (74, 89)
	Respiratory Rate, median (IQR)	18 (16, 18)
	Pulse Rate, median (IQR)	84 (74, 95)
Discharge destination		
	Discharged	2567 (60.7%)
	Transferred to ED	35 (0.8%)
	Transferred to ward	6 (0.1%)
	Unknown	1618 (38.3%)

Abbreviations: N, number; IQR, interquartile range; SPO2, oxygen saturation; ACEI, angiotensin-converting-enzyme inhibitor; ARB, angiotensin receptor blocker; GI, gastrointestinal; ED, emergency department.

eTable 2: Baseline characteristics of suspected cases

Factor	Swab not taken	Swab taken	p-value
N	1250	2976	
Age, years, median (IQR)	41 (28, 57)	39 (29, 53)	0.019
Sex - Male	577 (46.2%)	1091 (36.7%)	<0.001
Aboriginal and/or Torres Strait Islander	10 (0.8%)	31 (1.0%)	0.61
Comorbidities			
Cardiovascular disease	48 (3.8%)	107 (3.6%)	0.70
Diabetes	45 (3.6%)	86 (2.9%)	0.22
Hypertension	126 (10.1%)	265 (8.9%)	0.23
ACEI/ARB treatment	39 (3.1%)	100 (3.4%)	0.69
Smoking	136 (10.9%)	261 (8.8%)	0.032
Chronic renal or liver disease	16 (1.3%)	21 (0.7%)	0.067
Immunosuppressed	42 (3.4%)	91 (3.1%)	0.61
Chronic respiratory disease	112 (9.0%)	350 (11.8%)	0.008
Pregnancy	17 (1.4%)	39 (1.3%)	0.90
Overseas health facility exposure	9 (0.7%)	115 (3.9%)	<0.001
Australian health facility exposure	119 (9.5%)	911 (30.6%)	<0.001
Any contact or overseas travel	127 (10.2%)	1204 (40.5%)	<0.001
Contact with confirmed COVID-19 case	51 (4.1%)	516 (17.3%)	<0.001
Overseas travel (incl. cruise)	77 (6.2%)	738 (24.8%)	<0.001
Days from arrival to symptom onset, median (IQR)	5 (-1, 20)	2 (-1, 6)	0.016
Number of reported symptoms			<0.001
0	326 (26.1%)	50 (1.7%)	
1	92 (7.4%)	246 (8.3%)	
2	148 (11.8%)	548 (18.4%)	
3	178 (14.2%)	683 (23.0%)	
4	190 (15.2%)	650 (21.8%)	
5 or more	316 (25.3%)	799 (26.8%)	
Symptoms			
Any reported fevers	454 (36.3%)	1141 (38.3%)	0.22
Fever > 38°C	112 (9.0%)	277 (9.3%)	0.72
Fever subjective	374 (29.9%)	924 (31.0%)	0.47
Sore throat	615 (49.2%)	2066 (69.4%)	<0.001
Sinusitis	6 (0.5%)	14 (0.5%)	0.97
Cough	635 (50.8%)	2071 (69.6%)	<0.001
Shortness of breath	347 (27.8%)	909 (30.5%)	0.071
Chest pain	39 (3.1%)	72 (2.4%)	0.19
Anosmia	26 (2.1%)	76 (2.6%)	0.36
Ageusia	39 (3.1%)	81 (2.7%)	0.48
Anosmia or ageusia	52 (4.2%)	127 (4.3%)	0.87
Coryza	502 (40.2%)	1630 (54.8%)	<0.001
Diarrhoea	162 (13.0%)	491 (16.5%)	0.004
Other GI symptoms	29 (2.3%)	63 (2.1%)	0.68
Malaise/myalgia/arthralgia	479 (38.3%)	1422 (47.8%)	<0.001
Headache	137 (11.0%)	407 (13.7%)	0.016

Asymptomatic	315 (25.2%)	26 (0.9%)	<0.001
Days since symptom onset, median (IQR)	3 (2, 7)	3 (1, 6.5)	0.009
Clinical signs			
SPO2, median (IQR)	98 (97, 99)	98 (97, 99)	<0.001
Temperature Tympanic, median (IQR)	36.5 (36.2, 36.8)	36.6 (36.3, 36.9)	0.022
Systolic Blood Pressure, median (IQR)	132 (120, 146)	132.5 (121, 147)	0.48
Diastolic Blood Pressure, median (IQR)	81 (73, 88)	82 (75, 89)	0.010
Respiratory Rate, median (IQR)	18 (16, 18)	18 (16, 18)	0.51
Pulse Rate, median (IQR)	84 (74, 96)	84 (74, 94)	0.51
Discharge destination			<0.001
Discharged	639 (51.1%)	1928 (64.8%)	
Transferred to ED	15 (1.2%)	20 (0.7%)	
Transferred to ward	5 (0.4%)	1 (<0.1%)	
Unknown	591 (47.3%)	1027 (34.5%)	

Abbreviations: N, number; IQR, interquartile range; SPO2, oxygen saturation; ACEI, angiotensin-converting-enzyme inhibitor; ARB, angiotensin receptor blocker; GI, gastrointestinal; ED, emergency department.

eTable 3 – Positive and negative results for COVID-MATCH65

COVID-MATCH65 **Score**	**N (%) patients showing negative** COVID-MATCH65	**False negative** COVID-MATCH65, N (% negative)	**N (%) patients showing positive** COVID-MATCH65	**False positive** COVID-MATCH65, N (% positive)
≥0.5	1545 (36.6%)	4 (0.3%)	2681 (63.4%)	1844 (68.8%)
≥1	2095 (49.6%)	6 (0.3%)	2131 (50.4%)	1502 (70.5%)
≥1.5	2377 (56.2%)	8 (0.3%)	1849 (43.8%)	1378 (74.5%)
≥2	2927 (69.3%)	14 (0.5%)	1299 (30.7%)	948 (73.0%)
≥2.5	3145 (74.4%)	18 (0.6%)	1081 (25.6%)	812 (75.1%)
≥3	3624 (85.8%)	37 (1.0%)	602 (14.2%)	444 (73.8%)
≥3.5	3934 (93.1%)	60 (1.5%)	292 (6.9%)	197 (67.5%)
≥4	4062 (96.1%)	70 (1.7%)	164 (3.9%)	103 (62.8%)
≥4.5	4165 (98.6%)	84 (2.0%)	61 (1.4%)	31 (50.8%)

eTable 4: The sensitivity, specificity, positive predictive value and negative predictive value with 95% confidence intervals of various cut-off levels of COVID-MATCH65

Score	Sensitivity	Specificity	PPV	NPV	AUC
≥0.5	96.3 (90.8, 99.0)	34.8 (33.0, 36.6)	5.3 (4.4, 6.4)	99.6 (99.0, 99.9)	0.66 (0.64, 0.68)
≥1	94.4 (88.3, 97.9)	46.9 (45.0, 48.7)	6.4 (5.2, 7.7)	99.5 (99.0, 99.8)	0.71 (0.68, 0.73)
≥1.5*	92.6 (85.9, 96.7)	51.3 (49.4, 53.1)	6.8 (5.5, 8.2)	99.5 (98.9, 99.8)	0.72 (0.69, 0.75)
≥2	87.0 (79.2, 92.7)	66.5 (64.7, 68.2)	9.0 (7.4, 10.9)	99.3 (98.8, 99.6)	0.77 (0.73, 0.80)
≥2.5	83.3 (74.9, 89.8)	71.3 (69.6, 72.9)	10.0 (8.1, 12.1)	99.1 (98.6, 99.5)	0.77 (0.74, 0.81)
≥3	65.7 (56.0, 74.6)	84.3 (82.9, 85.6)	13.8 (10.9, 17.1)	98.5 (97.9, 98.9)	0.75 (0.70, 0.80)
≥3.5	44.4 (34.9, 54.3)	93.0 (92.0, 93.9)	19.6 (14.8, 25.1)	97.8 (97.1, 98.3)	0.69 (0.64, 0.73)
≥4	35.2 (26.2, 45.0)	96.4 (95.6, 97.0)	27.0 (19.8, 35.1)	97.5 (96.8, 98.0)	0.66 (0.61, 0.70)
≥4.5	22.2 (14.8, 31.2)	98.9 (98.4, 99.3)	43.6 (30.3, 57.7)	97.1 (96.4, 97.7)	0.61 (0.57, 0.65)

*Recommended cut-off

Abbreviations: PPV, positive predictive value; NPV, negative predictive value; AUC, area under the receiver operating characteristic curve

eTable 5: Univariate & multivariable analysis of features associated with a hospital admission

	**Univariate**		**Multivariate (AUC 0.85)**	
	OR (95% CI)	p	OR (95% CI)	p
Age, years	1.05 (1.01, 1.09)	0.021		
Male	4.65 (1.23, 17.57)	0.023	3.55 (0.85, 14.85)	0.083
Hypertension	4.97 (1.48, 16.66)	0.009		
Australian health facility exp	2.33 (0.55, 9.85)	0.249		
O/s travel or contact	0.35 (0.11, 1.19)	0.094	0.23 (0.06, 0.97)	0.046
Any fever	1.07 (0.36, 3.20)	0.902		
Coryza/sore throat	0.95 (0.30, 3.03)	0.934		
Cough	4.08 (0.51, 32.89)	0.186		
Shortness of breath	5.47 (1.74, 17.21)	0.004	5.29 (1.48, 18.94)	0.01
Anosmia/ageusia	0.80 (0.16, 3.91)	0.783		
Diarrhoea	1.71 (0.53, 5.57)	0.37		
Malaise/myalgia/arthralgia	2.31 (0.61, 8.75)	0.22		
Headache	1.04 (0.27, 4.08)	0.953		
SPO2 <97%	3.67 (1.19, 11.30)	0.024	3.68 (1.04, 13.06)	0.043
Temperature ≥37.5 C	1.43 (0.60, 3.43)	0.421		
Systolic blood pressure >140 mmHg	1.01 (0.33, 3.07)	0.987		
Diastolic blood pressure >80 mmHg	1.00 (0.31, 3.18)	1		
Respiratory rate <16/min or >20/min	2.71 (0.48, 15.43)	0.262		
Pulse rate <60/min or >100/min	1.44 (0.28, 7.40)	0.665		

eFigure 1: Participants assessed and tested in this study

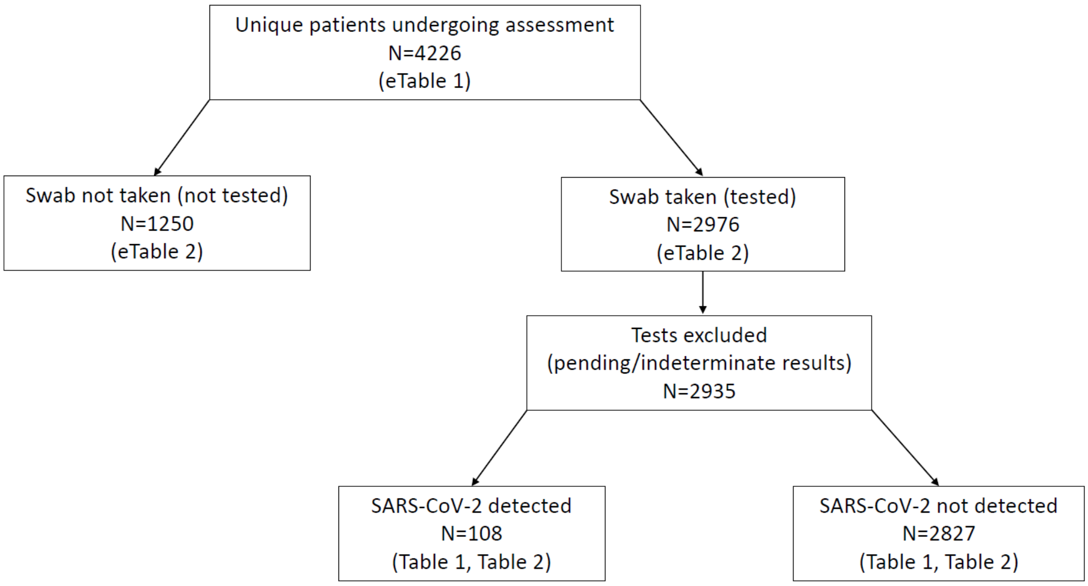

eFigure 2: Number of COVID-19 assessments performed per day

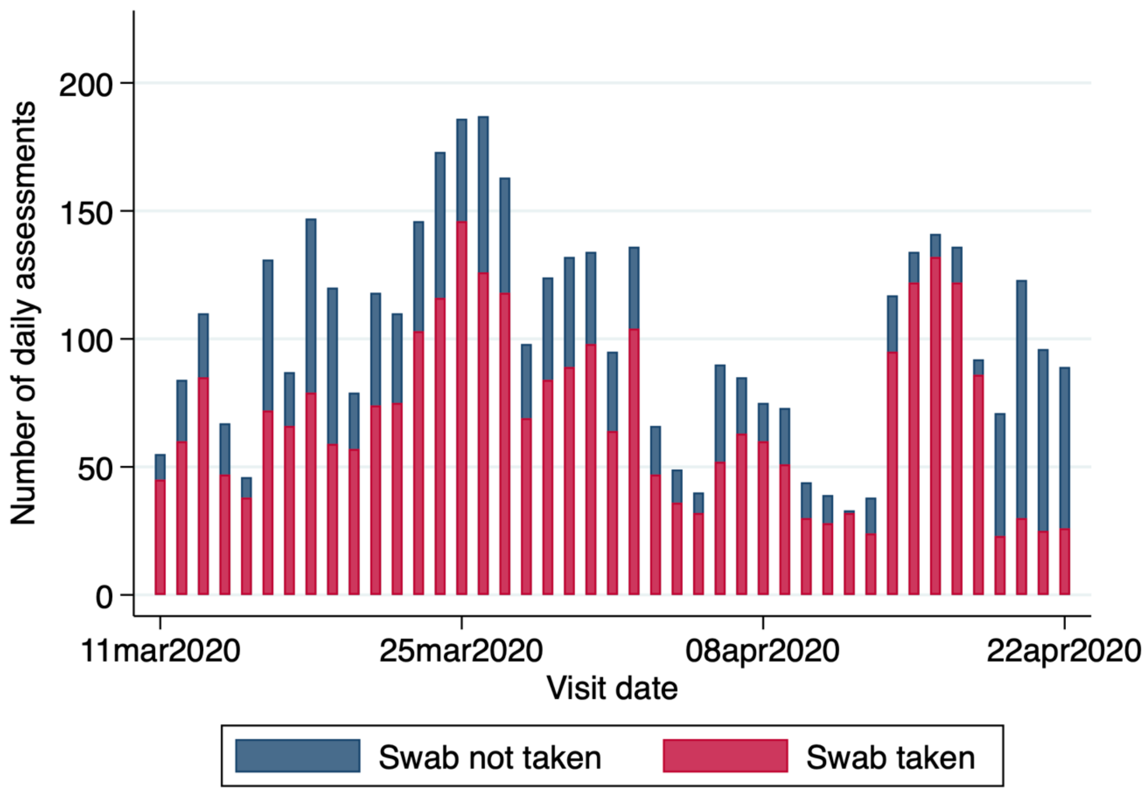

eFigure 3: Area under the receiver operating characteristic curve (AUC) analysis of COVID-MATCH65

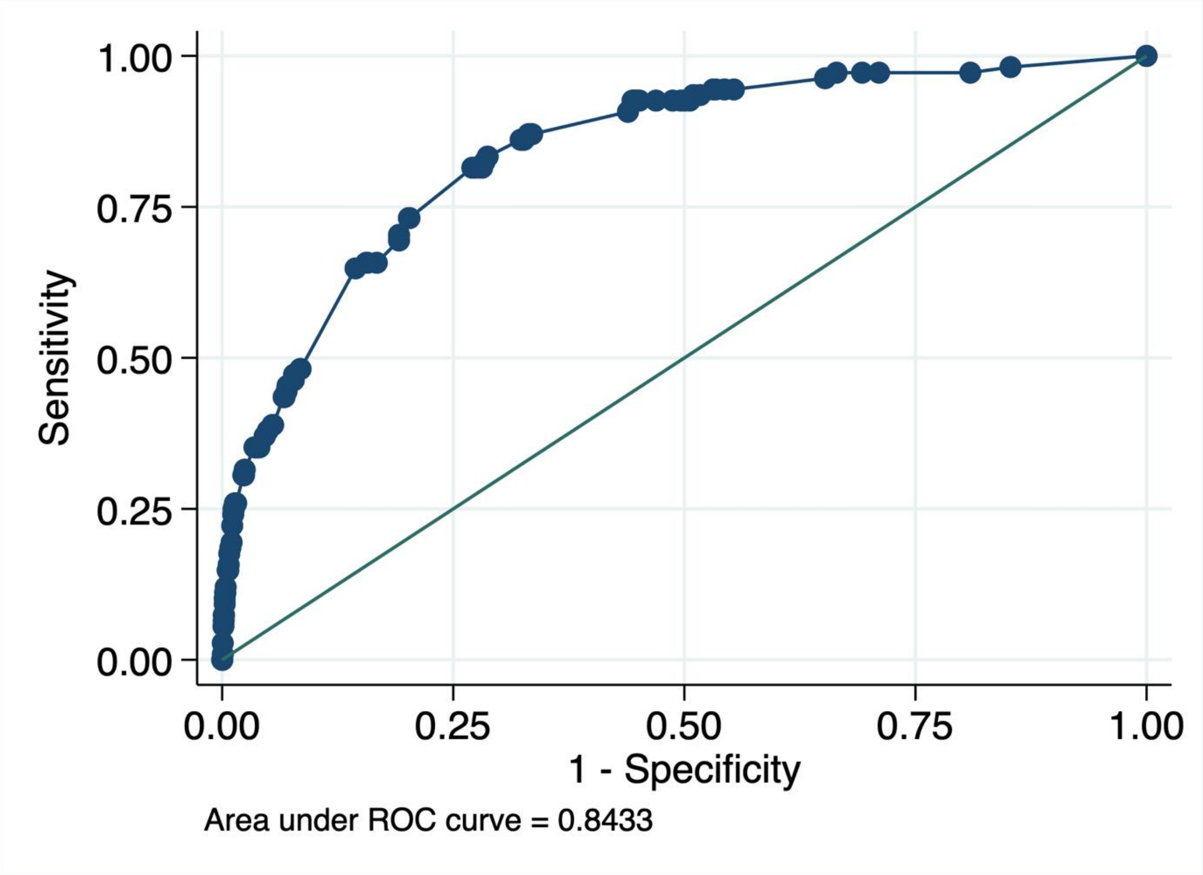

eFigure 4: Calibration of the COVID-MATCH65

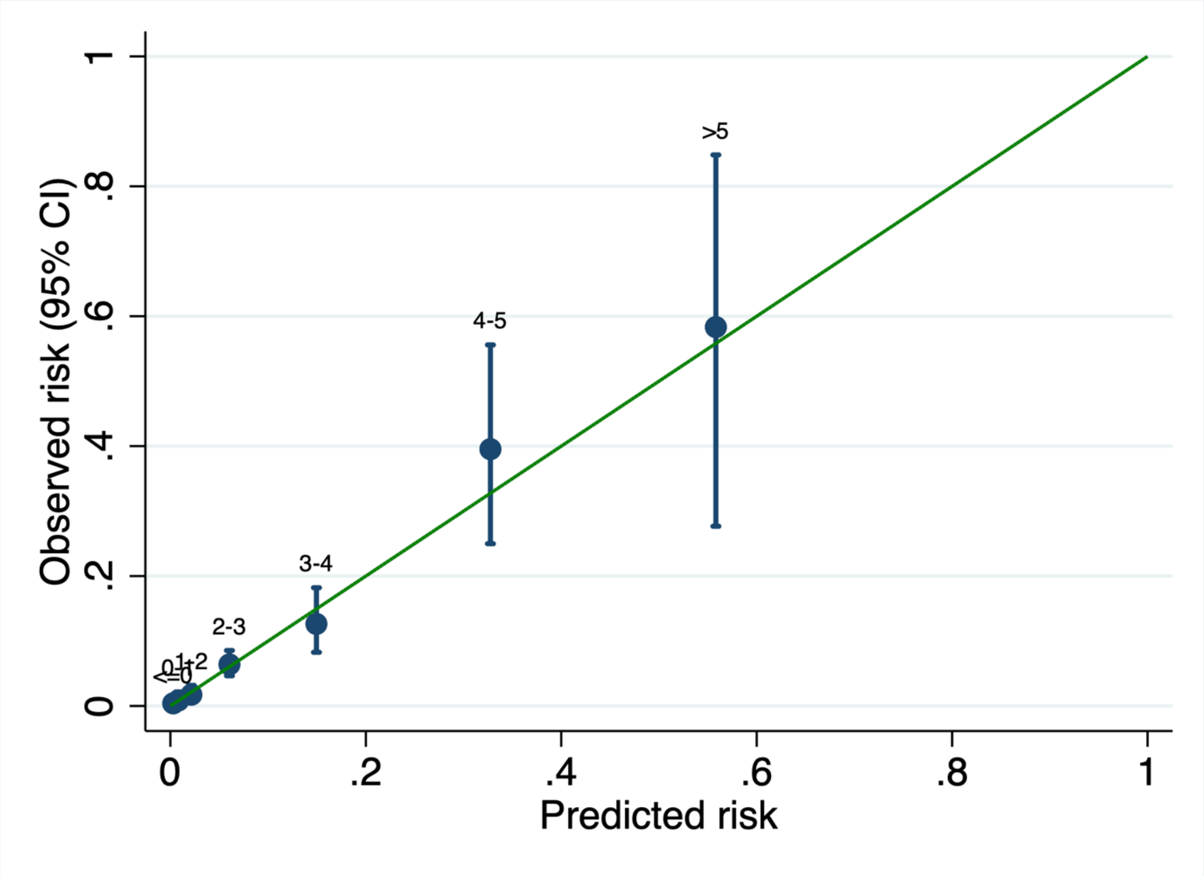

eFigure 3 Legend: Numbers above the bars represent the score ranges

References

Austin PC, Tu JV. Bootstrap Methods for Developing Predictive Models. The American Statistician 2004;58(2):7.

Corman VM, Landt O, Kaiser M, Molenkamp R, Meijer A, Chu DK, et al. Detection of 2019 novel coronavirus (2019-nCoV) by real-time RT-PCR. Euro Surveill 2020;25(3).

Steyerberg EW, Harrell FE, Jr., Borsboom GJ, Eijkemans MJ, Vergouwe Y, Habbema JD. Internal validation of predictive models: efficiency of some procedures for logistic regression analysis. Journal of clinical epidemiology 2001;54(8):774-81.
